# Supplementary material for: Bovine Fibroblast-Derived Extracellular Matrix Promotes the Growth and Preserves the Stemness of Bovine Stromal Cells during In Vitro Expansion
Source: J Funct Biomater. 2023 Apr 13;14(4):218. doi: 10.3390/jfb14040218 (PMC10144935; doi:10.3390/jfb14040218)
Supplement: Supplementary file 1 [file jfb-14-00218-s001.zip › jfb-2334371-supplementary.pdf]

Supplemental Materials (Lee K. et al.)

Supplemental Figure S1:

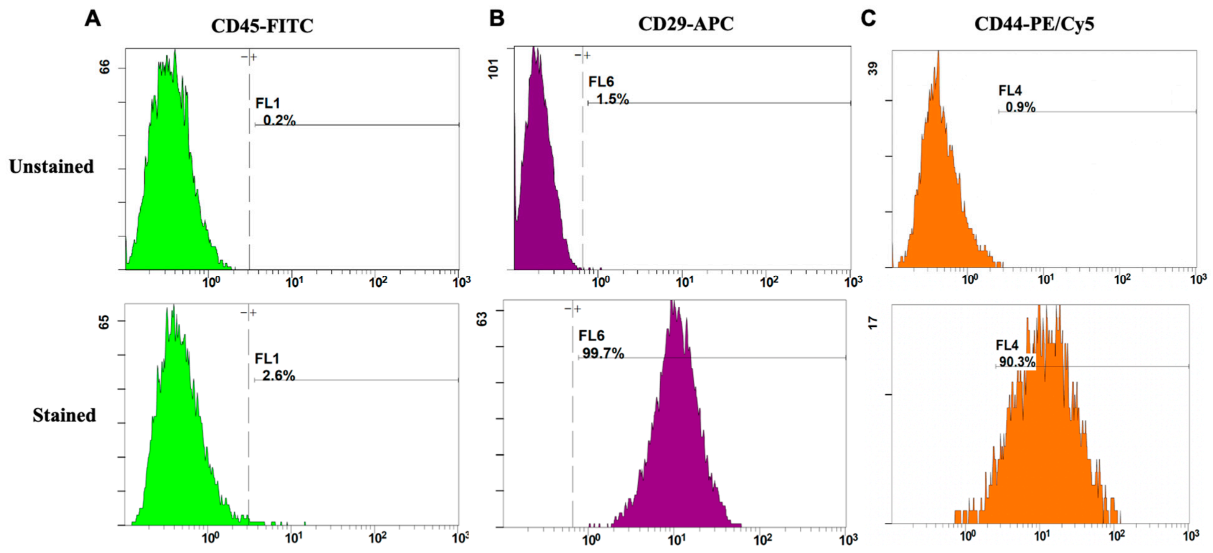

Figure S1. Flow cytometry analyses of BUSCs.
BUSCs in a 10 cm TCP dish were trypsinized using 1 mL of TrypLE (Cat# 12-605-010, Fisher Scientific). 4 mL of complete growth medium was added to the dish and mixed. Cells were counted and centrifuged at 1000rpm for 5 min in a Beckman Coulter centrifuge (ALR1L10, Beckman Coulter). Cells were resuspended to a Stain buffer (1x PBS +5% FBS +0.02% NaN3) at 2x10^6/mL. Cells were divided into two groups (one is for the unstained control, the other for the staining of antibodies). Antibodies were added to the cells and incubated in the dark at 4°C for 30 min. The antibodies used in the study are anti-CD45-FITC (Clone HI30, Cat#304006, Biolegend, CA), anti-CD29-APC (Clone TS2/16, Cat#303008, Biolegend, CA), anti-CD44-PE-Cy5 (CloneIM7, Cat#06511-70-100, Biogems, CA). The concentration of each antibody used in this study followed the manufacturers' instructions. After incubation with antibodies, cells were centrifuged at 4°C in an Eppendorf microcentrifuge at 400xg for 5 min (Brinkman Instruments). Cells were washed twice with PBS and then resuspended to the Stain buffer at (500k cells/300µL). Cells were then transferred to Falcon 5mL Round Bottom PP Test Tubes (Cat# 60819-728, VWR, PA) and analyzed using Gallios Flow Cytometer (Beckman Coulter) at Rutgers Flow Cytometry Core Facility.
(A) Flow cytometry analysis of BUSCs without staining (top) or with anti-CD45-FITC (bottom); (B) Flow cytometry analysis of BUSCs without staining (top) or with anti-CD29-APC (bottom); (C) Flow cytometry analysis of BUSCs without staining (top) or with anti-CD44-PE/Cy5 (bottom).

Supplemental Figure S2:

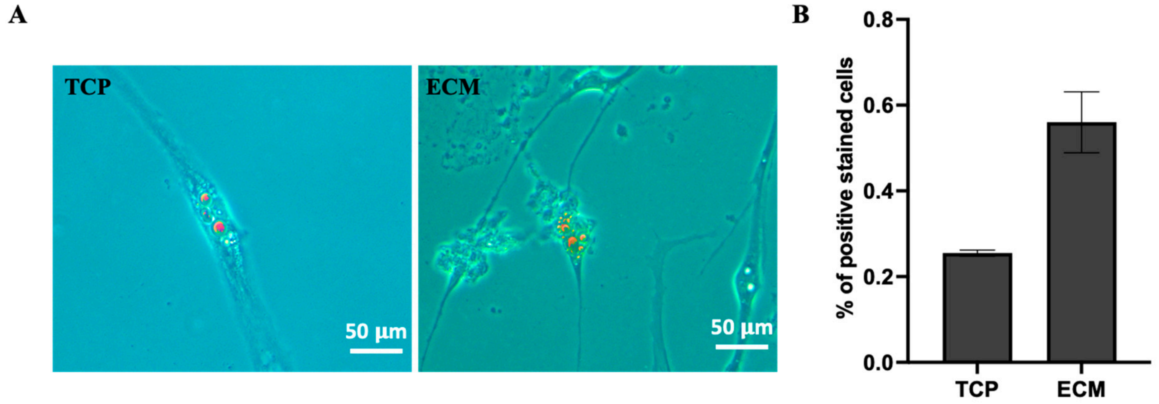

Figure S2. Adipogenic differentiation of BUSCs on TCP or BF-ECM with StemPro adipogenic medium. BUSCs at P4 were seeded on TCP or on BF-ECM (ECM), cultured and differentiated in the presence of StemPro adipogenic differentiation medium for 18 days and stained with Oil red O. (A) Representative images of Oil red O positively stained cells on TCP (left) and on BF-ECM (right). Scale bar = 50 µm. (B) Oil red O positively stained cells were counted and normalized to total number of cells. Data shown are average ± SD (n=2).
